# Adolescents’ Perspective Regarding a Community-Wide Mass Drug Administration Program for Soil-Transmitted Helminths in India

**DOI:** 10.4269/ajtmh.23-0676

**Published:** 2024-03-12

**Authors:** Kumudha Aruldas, Jabaselvi Johnson, Malvika Saxena, Angelin Titus, Amy Roll, Rohan Michael Ramesh, Judd L. Walson, Arianna Rubin Means, Sitara Swarna Rao Ajjampur

**Affiliations:** ^1^ The Wellcome Trust Research Laboratory, Division of Gastrointestinal Sciences, Christian Medical College, Vellore, India;; ^2^ Department of Global Health, University of Washington, Seattle, Washington;; ^3^ The DeWorm3 Project, University of Washington, Seattle, Washington;; ^4^ Departments of Global Health, Medicine, Pediatrics and Epidemiology, University of Washington, Seattle, Washington;; ^5^ Department of International Health, Johns Hopkins University, Baltimore, Maryland

## Abstract

This study was undertaken to understand the perspective of adolescents in endemic communities of India regarding soil-transmitted helminth (STH) infections and community-wide mass drug administration (cMDA). A multicountry community-based cluster-randomized trial, the Deworm3 trial, tested the feasibility of interrupting STH transmission with cMDA, where all individuals aged 1–99 are treated empirically with albendazole. Using a guideline based on the Consolidated Framework for Implementation Research, eight focus group discussions were conducted among 57 adolescents from the trial site in India and analyzed on ATLAS.ti 8.0 software using an a priori thematic codebook. Adolescents believed that adults could be a source of STH infection because they were not routinely dewormed like the children through the national deworming program. Perceived benefits of cMDA for all were better health and increased work efficiency. Perceived barriers to adults’ participation in cMDA was their mistrust about the program, fear of side effects, perceived low risk of infection, and absence during drug distribution. To encourage adult participation in cMDAs, adolescents suggested community outreach activities, engaging village influencers and health workers, and tailoring drug distribution to when adults would be available. Adolescents were confident in their ability to be change agents within their households for treatment compliance. Adolescents provided insights into potential barriers and solutions to improve adult participation in cMDA, identified best practices of cMDA delivery, and suggested that they have unique roles as change agents to increase their household participation in cMDA.

## INTRODUCTION

Soil-transmitted helminth (STH) caused by *Ascaris lumbricoides* (roundworm), *Trichuris trichiura* (whipworm), and *Ancylostoma duodenale* or *Necator americanus* (hookworms) infect 1.45 billion people worldwide with a high burden in India.^1^^–^^3^ A systematic review of STH surveys in children in India during 2000–2015 showed a wide variation in STH prevalence of *Ascaris* ranging from 0.6% to 91%, *Trichuris* from 0.7% to 72%, and hookworm from 0.02% to 52%.^4^ As per the WHO recommendations based on high prevalence (>50%), school-based biannual deworming programs have been implemented in India since 2015 as National Deworming Day (NDD) targeting children aged 1–19 years.^5^^,^^6^ A meta-analysis demonstrated that STH prevalence can “bounce back” after mass drug administration (MDA) to half or more of pre-MDA levels within 6 months and almost equal to pre-MDA levels within 12 months post-MDA.^7^ Mathematical modeling and other field studies indicate that expanding current school age targeted MDA to a community-wide mass drug administration (cMDA) approach may potentially interrupt STH transmission.^8^^–^^12^

To assess the feasibility of interrupting STH transmission with cMDA of albendazole, a multicountry community-based cluster-randomized trial (the DeWorm3 trial) was implemented in Benin, India, and Malawi.^13^^,^^14^ In addition to the clinical trial component, DeWorm3 included implementation science research to identify facilitating factors and barriers to cMDA implementation through a number of mixed-methods studies with stakeholders ranging from the community to ministries of health.^15^^–^^17^ The DeWorm3 study includes qualitative research from in-depth interviews with policymakers and focus group discussions (FGDs) with community members, including village leaders, men, women, and adolescents aged 10–15 years to identify barriers and facilitators to the implementation of STH cMDA programs.^18^^–^^20^ In this article, we present the results of the FGDs conducted among adolescents in India to understand their perspectives on STH infections, modes of spread and prevention, challenges, and possible solutions in implementing STH cMDA program with high coverage with specific reference to adult community members.

## MATERIALS AND METHODS

### Study location and setting.

**Figure 1.**
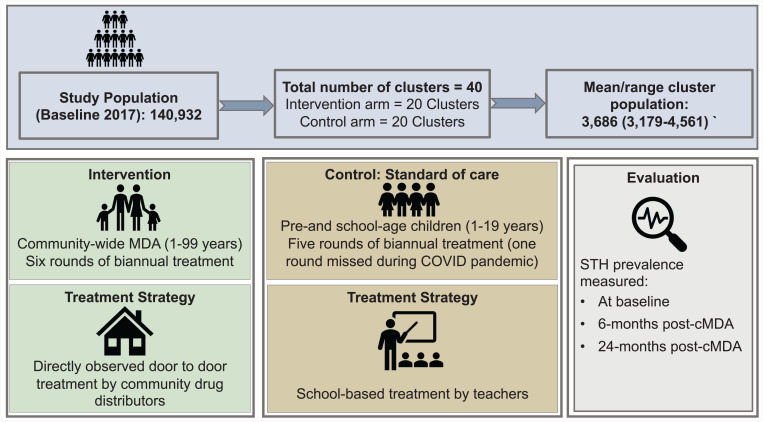
DeWorm3 study design. cMDA = community-wide mass drug administration.

The DeWorm3 trial in India was implemented in two blocks of Tamil Nadu-Timiri block in Ranipet district (formerly Vellore district) and Jawadhu Hills block in Tiruvannamalai district. The trial area covered a population of 140,932 residing in 36,536 households across 219 villages in Timiri and 154 villages in Jawadhu Hills.^21^ The area includes 77% rural and 23% tribal (disadvantaged communities or groups of people listed in a schedule of the Indian constitution) population wherein more than 90% were involved in agricultural activities and only 34.6% of the households had access to improved sanitation.^21^ The trial area was demarcated into 20 intervention and 20 control clusters based on administrative and geographic boundaries with an average cluster population of 3,686 (range: 3,170–4,561) (Figure 1). A household identification card with the name and address of the head of the household and household identification number and barcode was issued to all households in the trial area. The baseline STH prevalence among all individuals older than 1 year, conducted between December 2017 and February 2018, was 21.4%.^21^ In the intervention clusters, all individuals aged 1–99 years were included for deworming by the trained community drug distributors of the DeWorm3 trial, whereas in the control clusters, children aged 1–19 years were dewormed through the school-based NDD program of the Government of India, the standard of care.

### Data collection and analysis.

Eight focus group discussions (FGDs) were conducted among school-age adolescents between 10 and 15 years of age residing in the intervention clusters. These FGDs were conducted at two time points, four FGDs during January and February 2018 before the first round of cMDA in March 2018 (formative qualitative research) and four FGDs in March 2019 immediately after the third round of cMDA in February 2019 (midline qualitative research). A multistage sampling technique was used to select the study participants. In the formative research, four intervention clusters were randomly selected, and then the participants were purposively selected from the census list in each of the four clusters. In the midline qualitative research, the intervention clusters were divided into high- and low-treatment coverage based on the median treatment coverage of the previous two cMDAs, and then four clusters (two above and two below the median) were randomly selected. Adolescents living within 3–5 km of the venue for FGD in each cluster were randomly selected from the DeWorm3 trial census list.

The focus of the FGDs was on the inclusion of adults in the deworming program and not just the children alone. A semistructured interview guide was developed based on the Consolidated Framework for Implementation Research (CFIR), which is a meta-theoretical determinants framework used to identify barriers and facilitators to implementation.^22^ This framework consists of 38 constructs under five domains: 1) innovation characteristics, 2) outer setting, 3) inner setting, 4) characteristics of individuals, and 5) process. In addition, the study team identified three non-CFIR constructs—STH knowledge, DeWorm3 trial conduct, and equity—a priori to use in the analysis. Social scientists trained in qualitative data collection techniques and the interview guide conducted the FGDs along with the notetakers. The FGDs were in the local language (Tamil), recorded on digital voice recorders, transcribed, and translated into English. Two primary coders, trained on the a priori thematic codebook, coded the English transcripts independently on ATLAS.ti 8.0 software (Scientific Software Development, Berlin, Germany). The coding was finalized primarily in consensus between the two coders but included a third coder designated as the “tie-breaker” when necessary.

## RESULTS

A total of 57 adolescents, 34 in formative research and 23 in midline research, participated in the FGDs. Except for one, all were enrolled in school at the time of data collection, and their median age was 13.6 years. A total of 21 girls and 36 boys participated, and the girl–boy ratio was similar in the formative (1:1.8) and midline (1:1.6) FGDs. The analysis resulted in eight CIFR themes (design quality and packaging, complexity, adaptability, relative advantage, patient needs and resources, engaging innovation participants and opinion leaders, and knowledge and beliefs about the intervention) influencing four domains (innovation characteristics, characteristics of individuals, outer setting, and process) emerged from the analysis. The key findings of their awareness and beliefs about STH, perceptions, and opinions on cMDA implementation including barriers and solutions to participation in cMDA, and STH elimination are presented in this section.

### Adolescents were aware of STH infections and believed adults in their communities would be STH infected.

The adolescents who participated in the formative research had learned about STH infections from various sources including their teachers during drug distribution at school, school textbook chapters about worms, doctors when they had sought treatment for abdominal pain, banners in the hospital, or they had noticed worms in their stool. A few of them related STH to earthworms and worms in fruits. They believed that the adults in their communities would be infected with STH because they engage in agricultural work barefoot, are likely to be walking in open defecation areas while farming, and, unlike children treated for STH in schools, adults were not. One of them said,

*Suppose there is a person who is working in the field and ploughs…. they can’t work with slippers in the mud. They just get down and work as it is (without footwear). Whether they go (for defecation), … they will be stepping everywhere. If they go to bathroom, urinate, or defaecate, whatever it may be, they will be stepping on it, so it (intestinal worms) will be coming to them.* (Baseline FGD, Cluster 17)

They described STH spread by not washing hands, walking barefoot, open defecation, playing in the mud, and eating food on which houseflies would have sat. They believed that the adults could spread STH infections to their children while feeding them or sharing the same plate while eating without washing their hands. They discussed that STH infections cause abdominal pain, loss of appetite, and lethargy, which could be prevented by keeping the nails trimmed and clean, washing hands before eating and after using the toilet, avoiding food on which houseflies could have sat, wearing footwear, and using toilets.

### Despite the perceived benefits of STH cMDA, the adolescents were concerned about adult participation in cMDA.

In the formative research, the adolescents expressed that through cMDA, the adults would be free from symptoms of STH infections and thus have health benefits and increased work efficiency. However, they were concerned that adults would be worried about the side effects of the deworming tablets, such as diarrhea, vomiting, leg pain, headache, nausea, stomach pain, allergy, and fever. They particularly noted that adults on treatment for other diseases and those who think they do not have worms would be hesitant to accept deworming treatment. Further, they felt that adults would generally refuse to participate in cMDA if unaware of the purpose of tablet distribution and unfamiliar persons distribute the tablets.

*… they (adults) will ask questions—where are you coming from? … Who are you? … they will ask, from where did they get (the tablet) and how did you get this tablet? … what is this tablet for? … no one will take and eat it … they will ask who gave this … which place you are from, how did you come in search of our house?* (Baseline FGD, Cluster 12)

**Figure 2.**
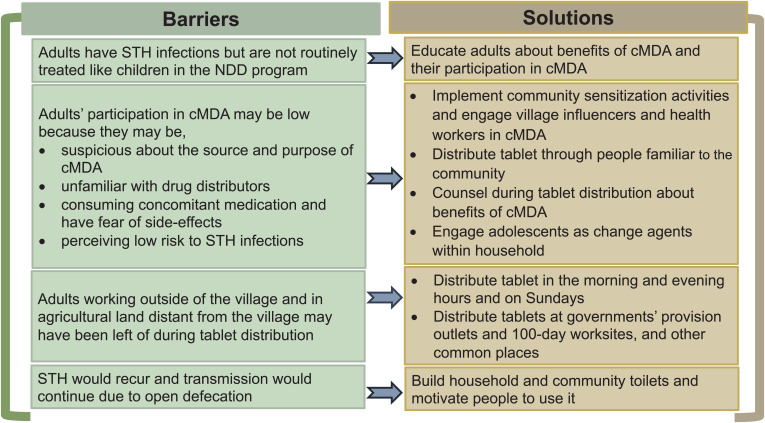
Barriers and solutions to community-wide mass drug administration (cMDA) delivery as perceived by the adolescents. NDD = National Deworming Day; STH = soil-transmitted helminth.

On the basis of their observation of how activities were generally organized in villages, the adolescent participants suggested informing the community about the benefits of cMDA through village leaders, including ward members, the vice president, councilors, and the secretary of the villages, and by various community mobilization activities such as making announcements about the program using a loudspeaker or *damukku* (drum beating), putting up banners, and posting notices in the communities (Figure 2). They gave emphasis to house visits for information sharing as adults would pay greater attention when spoken to face-to-face. They said that instead of compelling people to eat the tablet, the benefits of the tablet should be explained to them patiently. They believed the treatment would be better accepted if government village health nurses and *anganwadi* workers (government preschool workers) informed the community members and distributed at a time convenient to the adults. The adolescents suggested giving information about deworming approximately 2–3 days before distribution or people would forget about it. A few of them were confident of convincing their parents to consume the deworming tablets because of their higher literacy status compared with their parents.

*… they (adults) will call any educated child and ask them about what kind of tablet it is? … now if we go and tell in our house about what we spoke here, if we tell our mother and father, little by little they will understand that the worms in the stomach will be eliminated… . if we say like that our mother and father will accept.* (Baseline FGD, Cluster 12)

### Adolescents perceived challenges in controlling STH infections.

The adolescents who participated in the formative research believed that one-time treatment would be inadequate to cure STH infections, particularly among those having a high worm load. They perceived that it might not be possible to eliminate STH infections because STH infections would spread from those who did not eat the tablet or were not available to receive the tablet during distribution. They believed that even if STH was controlled by distributing tablets to everyone in the community, it might recur from the soil because of open defecation. Participants believed that control of STH infections would be possible if everyone built toilets and used them.

*Somehow it keeps spreading…. We can’t destroy it completely. While you are giving tablet, if someone is not here in this village that tablet will not go and reach them …, if they come here, they will have intestinal worm in their stomach, if they pass stools here and there and if people who ate the tablet step on it, worm will form in their stomach.* (Baseline FGD, Cluster 17)

### Adolescents identified the best practices of and gaps in cMDA implementation.

**Figure 3.**
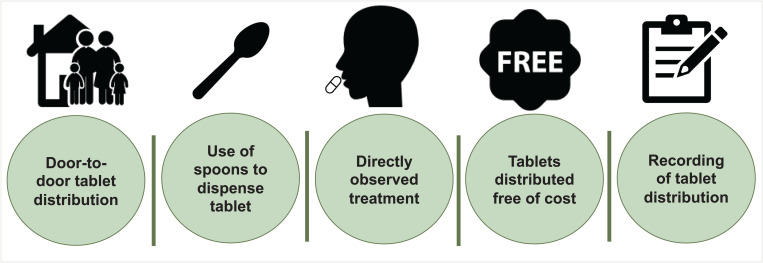
Best practices of DeWorm3 community-wide mass drug administration implementation as identified by the adolescents.

Adolescent participants in the midline FGDs conducted after three rounds of cMDA reported positive experiences of their family members being cured of symptoms such as stomach pain, poor appetite, and worms in stool after cMDA. They highly appreciated that the drug distributors went door-to-door to distribute tablets, which was helpful for people, particularly the elderly; used spoons to dispense the tablets hygienically; ensured that people consumed their tablets in front of them; distributed tablets free of cost; and made notes of who received and who did not receive the tablet (Figure 3).

One of them said:

*because they [people] throw it [the tablet] away, they are asking to eat in front of their [drug distributors’] eyes. … they brought tablet in a box and gave with spoon … even if the person is not available, they come later and give. … They would have not got if they had gone for work in the field … in another place.* (Midline FGD, Cluster 13)

They had noted drug distributors verifying the Deworm3 household identification card, giving drugs only to those present in the house, enquiring about the availability of family members absent during tablet distribution, making revisits to give the tablet, and going to those working in agricultural fields to distribute tablets. The adolescents were correctly aware that children younger than 1 year and those who had consumed alcohol were excluded from treatment. Some believed that some adults may not have wanted to swallow the tablet and may have thrown it away, and some adults working in agricultural fields and outside the village may have missed receiving the tablet.

*… they asked to bring the card [household ID card] …. they had a name list … and if they find their name, they give …. they saw the person with their own eyes and gave … near my house more than 10 houses are there, they came all the way and gave the tablet … they came and enquired even one week later and gave (tablet).* (Midline FGD, Cluster 34)

## DISCUSSION

This qualitative research showed that adolescents are aware of STH infections, modes of transmission, and prevention measures. However, they have similar misconceptions as adults in the same community, such as conflating STH with earthworms and worms in fruits.^19^ They believed that adults would be STH-infected and be a source of infection because they were not routinely dewormed like the preschool and school-age children and that they would benefit if dewormed. These adolescents living in communities with unimproved sanitation facilities and engaged in farming were uncertain about the elimination of STH infections due to the persisting practice of open defecation in their communities.^21^

The participants of this study identified several barriers to adults’ participation in STH cMDA, such as adults’ mistrust of cMDA programs; fear of side effects of deworming, specifically if they are on concomitant medication; their perceived low risk to STH infections; and their absence during drug distribution, similar to barriers reported by the adults in MDA programs for STH and lymphatic filariasis (LF) in India, Benin, Malawi, and Tanzania.^20^^,^^23^^–^^26^ Similarly, as suggested in other studies, the adolescents in this study believed that community outreach activities, involvement of village leaders and government health workers, treatment delivery by trusted drug distributors at a time and day convenient to the community members, and counseling during drug distribution would facilitate adult participation in cMDA.^27^

Free door-to-door treatment delivery, directly observed treatment, using spoons to dispense tablets, recording treatment details, and revisiting those who missed treatment were considered the best practices of cMDA delivery by the adolescents of this study. Literature from Sri Lanka and Kenya on LF MDA studies shows that door-to-door treatment was appreciated by community members even in an urban setting and considered the best strategy to increase treatment coverage.^28^^,^^29^ A review of factors influencing compliance and interviews of experts with experience implementing MDA showed that directly observed therapy has an impact on compliance with MDA.^30^^,^^31^ The adolescents of other LF MDA studies in southern India had also perceived that low treatment coverage was because of drug distributors not revisiting houses.^26^

The adolescents in this study were confident that they could ensure treatment compliance among the adults in their household. The adolescents have been effective change agents in various programs such as water, sanitation, and hygiene programs in Zambia and cardiovascular risk factors and weight loss in Sri Lanka.^32^^–^^34^ Voices of adolescents are considered vital in evaluation efforts because they are often well informed about programs in their communities and may also be the participants of program and research issues.^35^^,^^36^ It is feasible to reach adolescents with information about cMDA in India on NDD when they are gathered in schools to receive albendazole and at the *anganwadi* centers in their communities for nutrition and health programs.^5^^,^^37^

The major limitation of this study is that the gender-specific analysis to understand the agreements and disagreements in observations and perceptions of adolescent boys and girls was not possible. Similarly, there may have been differences in opinion between adolescents enrolled in and out of school, and this study almost exclusively engaged adolescents enrolled in school. Although this study provides valuable insights into how adolescents perceived STH cMDA programs and the role they can play in advocating for such programs, further research would be needed to understand the impact of actively engaging adolescents in cMDA on treatment coverage and compliance. The results of this study are context-specific and may not be generalizable because they may vary depending on cultural differences, especially in the urban–rural context in India.

In conclusion, the adolescents in this study provided valuable insights into barriers, facilitating factors, and best practices of the STH cMDA program. Given that adolescents between 10 and 19 years constitute a considerable section of India’s population (253 million), their involvement in future STH cMDA programs could be explored for information sharing, ensuring treatment compliance, and obtaining quick feedback about the program implementation.^38^

## References

[1] World Health Organization , 2017. *Guideline: Preventive Chemotherapy to Control Soil-Transmitted Helminth Infections in at-Risk Population Groups*. Available at: http://www.ncbi.nlm.nih.gov/books/NBK487927/. Accessed May 7, 2021. 29578660

[2] Pullan RL Smith JL Jasrasaria R Brooker SJ, 2014. Global numbers of infection and disease burden of soil transmitted helminth infections in 2010. Parasit Vectors 7: 37. 24447578 10.1186/1756-3305-7-37 PMC3905661

[3] Lai YS Biedermann P Shrestha A Chammartin F à Porta N Montresor A Mistry NF Utzinger J Vounatsou P, 2019. Risk profiling of soil-transmitted helminth infection and estimated number of infected people in South Asia: A systematic review and Bayesian geostatistical analysis. PLoS Negl Trop Dis 13: e0007580. 31398200 10.1371/journal.pntd.0007580 PMC6709929

[4] Salam N Azam S, 2017. Prevalence and distribution of soil-transmitted helminth infections in India. BMC Public Health 17: 201. 28209148 10.1186/s12889-017-4113-2 PMC5311856

[5] Government of India , 2015. *National Deworming Day: Operational Guidelines*. Available at: https://www.nhp.gov.in/national-deworming-day_pg. Accessed January 22, 2021.

[6] World Health Organization , 2012. *Soil-Transmitted Helminthiases: Eliminating Soil-Transmitted Helminthiases as a Public Health Problem in Children: Progress Report 2001–2010 and Strategic Plan 2011–2020*. Available at: https://iris.who.int/bitstream/handle/10665/44804/9789241503129_eng.pdf?sequence=1. Accessed January 22, 2021.

[7] Jia TW Melville S Utzinger J King CH Zhou XN, 2012. Soil-transmitted helminth reinfection after drug treatment: A systematic review and meta-analysis. PLoS Negl Trop Dis 6: e1621. 22590656 10.1371/journal.pntd.0001621 PMC3348161

[8] Anderson RM Truscott JE Pullan RL Brooker SJ Hollingsworth TD, 2013. How effective is school-based deworming for the community-wide control of soil-transmitted helminths? PLoS Negl Trop Dis 7: e2027. 23469293 10.1371/journal.pntd.0002027 PMC3585037

[9] Truscott J Turner H Anderson R, 2015. What impact will the achievement of the current World Health Organisation targets for anthelmintic treatment coverage in children have on the intensity of soil transmitted helminth infections? Parasit Vectors 8: 551. 26490544 10.1186/s13071-015-1135-4 PMC4618937

[10] Brooker SJ Nikolay B Balabanova D Pullan RL, 2015. Global feasibility assessment of interrupting the transmission of soil-transmitted helminths: A statistical modelling study. Lancet Infect Dis 15: 941–950. 25886799 10.1016/S1473-3099(15)70042-3

[11] Pullan RL , 2019. Effects, equity, and cost of school-based and community-wide treatment strategies for soil-transmitted helminths in Kenya: A cluster-randomised controlled trial. Lancet 393: 2039–2050. 31006575 10.1016/S0140-6736(18)32591-1 PMC6525786

[12] Clarke NE Clements ACA Doi SA Wang D Campbell SJ Gray D Nery SV, 2017. Differential effect of mass deworming and targeted deworming for soil-transmitted helminth control in children: A systematic review and meta-analysis. Lancet 389: 287–297. 27979381 10.1016/S0140-6736(16)32123-7

[13] The DeWorm3 Trials Team , 2020. Baseline patterns of infection in regions of Benin, Malawi and India seeking to interrupt transmission of soil transmitted helminths (STH) in the DeWorm3 trial. PLoS Negl Trop Dis 14: e0008771. 33137100 10.1371/journal.pntd.0008771 PMC7673551

[14] Ásbjörnsdóttir KH , 2018. Assessing the feasibility of interrupting the transmission of soil-transmitted helminths through mass drug administration: The DeWorm3 cluster randomized trial protocol. PLoS Negl Trop Dis 12: e0006166. 29346377 10.1371/journal.pntd.0006166 PMC5773085

[15] Means AR , 2018. Evaluating the sustainability, scalability, and replicability of an STH transmission interruption intervention: The DeWorm3 implementation science protocol. PLoS Negl Trop Dis 12: e0005988. 29346376 10.1371/journal.pntd.0005988 PMC5773078

[16] Gwayi-Chore MC , 2022. Defining optimal implementation packages for delivering community-wide mass drug administration for soil-transmitted helminths with high coverage. BMC Health Serv Res 22: 792. 35717193 10.1186/s12913-022-08080-5 PMC9206125

[17] Means AR , 2021. Structural readiness to implement community-wide mass drug administration programs for soil-transmitted helminth elimination: Results from a three-country hybrid study. Implement Sci Commun 2: 80. 34281614 10.1186/s43058-021-00164-3 PMC8287777

[18] Roll A , 2022. Policy stakeholder perspectives on barriers and facilitators to launching a community-wide mass drug administration program for soil-transmitted helminths. Glob Health Res Policy 7: 47. 36461087 10.1186/s41256-022-00281-z PMC9716752

[19] Aruldas K , 2020. Gender differences in the perceived need for community-wide deworming: Formative qualitative research from the DeWorm3 study, India. PLoS Negl Trop Dis 14: e0008829. 33237928 10.1371/journal.pntd.0008829 PMC7688162

[20] Avokpaho E , 2022. It depends on how you tell: A qualitative diagnostic analysis of the implementation climate for community-wide mass drug administration for soil-transmitted helminth. BMJ Open 12: e061682. 10.1136/bmjopen-2022-061682 PMC9198697 35701056

[21] Ajjampur SSR , 2021. Epidemiology of soil transmitted helminths and risk analysis of hookworm infections in the community: Results from the DeWorm3 Trial in southern India. PLoS Negl Trop Dis 15: e0009338. 33930024 10.1371/journal.pntd.0009338 PMC8184002

[22] Damschroder LJ Aron DC Keith RE Kirsh SR Alexander JA Lowery JC, 2009. Fostering implementation of health services research findings into practice: A consolidated framework for advancing implementation science. Implement Sci 4: 50. 19664226 10.1186/1748-5908-4-50 PMC2736161

[23] Murphy E Togbevi IC Ibikounlé M Avokpaho EF Walson JL Means AR, 2023. Soil-transmitted helminth surveillance in Benin: A mixed-methods analysis of factors influencing non-participation in longitudinal surveillance activities. PLoS Negl Trop Dis 17: e0010984. 36626399 10.1371/journal.pntd.0010984 PMC9831304

[24] Babu BV Kar SK, 2024. Coverage, compliance and some operational issues of mass drug administration during the programme to eliminate lymphatic filariasis in Orissa, India. Trop Med Int Health 9: 702–709. 10.1111/j.1365-3156.2004.01247.x 15189460

[25] Kisoka WJ Simonsen PE Malecela MN Tersbøl BP Mushi DL Meyrowitsch DW, 2014. Factors influencing drug uptake during mass drug administration for control of lymphatic filariasis in rural and urban Tanzania. PLoS One 9: e109316. 25296034 10.1371/journal.pone.0109316 PMC4190414

[26] Hussain MA Sitha AK Swain S Kadam S Pati S, 2014. Mass drug administration for lymphatic filariasis elimination in a coastal state of India: A study on barriers to coverage and compliance. Infect Dis Poverty 3: 31. 25237478 10.1186/2049-9957-3-31 PMC4166397

[27] Aruldas K , 2023. Evaluation of opportunities to implement community-wide mass drug administration for interrupting transmission of soil-transmitted helminths infections in India. PLoS Negl Trop Dis 17: e0011176. 36897877 10.1371/journal.pntd.0011176 PMC10004831

[28] Weerasooriya MV Yahathugoda CT Wickramasinghe D Gunawardena KN Dharmadasa RA Vidanapathirana KK Weerasekara SH Samarawickrema WA, 2007. Social mobilisation, drug coverage and compliance and adverse reactions in a mass drug administration (MDA) programme for the elimination of lymphatic filariasis in Sri Lanka. Filaria J 6: 11. 18005398 10.1186/1475-2883-6-11 PMC2203982

[29] Njomo DW Mukoko DA Nyamongo NK Karanja J, 2014. Increasing coverage in mass drug administration for lymphatic filariasis elimination in an urban setting: A study of Malindi Town, Kenya. PLoS One 9: e83413. 24454703 10.1371/journal.pone.0083413 PMC3891599

[30] Krentel A Fischer PU Weil GJ, 2013. A review of factors that influence individual compliance with mass drug administration for elimination of lymphatic filariasis. PLoS Negl Trop Dis 7: e2447. 24278486 10.1371/journal.pntd.0002447 PMC3836848

[31] Newby G , 2015. Review of mass drug administration for malaria and its operational challenges. Am J Trop Med Hyg 93: 125–134. 26013371 10.4269/ajtmh.14-0254 PMC4497884

[32] Bresee S Caruso BA Sales J Lupele J Freeman MC, 2016. “A child is also a teacher”: Exploring the potential for children as change agents in the context of a school-based WASH intervention in rural Eastern Zambia. Health Educ Res 31: 521–534. 27206442 10.1093/her/cyw022

[33] Chandraratne N Yamaguchi M Indrawansa S Gunawardena N Kuwahara K Islam Z Kawasaki Y Mizoue T Samarasinghe D, 2019. The effect of youths as change agents on cardiovascular disease risk factors among adult neighbours: A cluster randomised controlled trial in Sri Lanka. BMC Public Health 19: 893. 31286931 10.1186/s12889-019-7142-1 PMC6613264

[34] Gunawardena N Kurotani K Indrawansa S Nonaka D Mizoue T Samarasinghe D, 2016. School-based intervention to enable school children to act as change agents on weight, physical activity and diet of their mothers: A cluster randomized controlled trial. Int J Behav Nutr Phys Act 13: 45. 27048282 10.1186/s12966-016-0369-7 PMC4822262

[35] Powers JL Tiffany JS, 2006. Engaging youth in participatory research and evaluation. J Public Health Manag Pract 12: S79–S87. 10.1097/00124784-200611001-00015 17035908

[36] Sabo K, 2001. *The Benefits of Participatory Evaluation for Children and Youth*. Available at: https://www.iied.org/sites/default/files/pdfs/migrate/G01967.pdf. Accessed February 20, 2023.

[37] Government of India, Ministry of Health & Family Welfare , 2018. *Implementation Guidelines: Rashtriya Kishore Swasthya Karyakram (RKSK).* Available at: https://nhm.gov.in/New_Updates_2018/NHM_Components/RMNCHA/AH/guidelines/Implementation_Guidelines_Rashtriya_Kishor_Swasthya_Karyakram(RKSK)_2018.pdf. Accessed February 20, 2023.

[38] Government of India, Ministry of Health & Family Welfare , 2014. *Rashtriya Kishor Swasthya Karyakram: Operational Framework.* Available at: https://nhm.gov.in/images/pdf/programmes/RKSK/RKSK_Operational_Framework.pdf. Accessed January 5, 2023.

